# New fossil data reveal evolutionary pathways within the genus *Trichoneura* Loew, 1850 (Diptera, Limoniidae)

**DOI:** 10.1038/s41598-023-43468-1

**Published:** 2023-10-05

**Authors:** Katarzyna Kopeć, Iwona Kania-Kłosok, Qingqing Zhang, Wiesław Krzemiński

**Affiliations:** 1 Institute of Systematics and Evolution of Animals, Polish Academy of Sciences, Kraków, Poland; 2 Institute of Biology, University of Rzeszów, Rzeszów, Poland; 3 State Key Laboratory of Palaeobiology and Stratigraphy, Nanjing Institute of Geology and Palaeontology, Chinese Academy of Sciences, No. 39, East Beijing Road, Nanjing, 210008 People’s Republic of China

**Keywords:** Evolution, Environmental sciences

## Abstract

New inclusions of *Trichoneura* preserved in Upper Cretaceous (Cenomanian) Kachin amber allow the description of a new subgenus, *Burmania* subgen. nov., and four new species: *Trichoneura* (*Burmania*) *burmitensis* subgen. et sp. nov., *Trichoneura* (*Burmania*) *chungkuni* subgen. et sp. nov., *Trichoneura* (*Burmania*) *sevciki* subgen. et sp. nov. and *Trichoneura* (*Burmania*) *wangi* subgen. et sp. nov. The species differ mainly by the morphology of the hypopygium or wing venation but also the construction of the antenna. Based on a comparison of the wing venation and the morphology of the hypopygium it was possible to describe features which are characteristic of the new subgenus, especially the presence of vein R_3+4_. Moreover, it was possible to elucidate the evolutionary pattern of *Trichoneura* with two distinct extant and extinct branches. *Trichoneura* (*Trichoneura*) *canadensis* from Upper Cretaceous Canadian amber is transferred to the new subgenus *Burmania*.

## Introduction

Diptera appeared probably in the Triassic, as indicated by the fossil record. The oldest representative, *Grauvogelia arzvilleriana* Krzemiński, Krzemińska and Papier^1^, is known from sediments, and was described based on a fossilized wing. The presence of Nematocera in the Triassic fauna is confirmed by inclusions in amber from Italy (the deposits were discovered in the Alps), in which a specimen of a nematoceran fly was found of unspecified taxonomic position^2^. In the Mesozoic era, there was an evolutionary radiation, which led to increased diversity of Diptera. The oldest representatives of the Tipulomorpha belong to the family Archilimoniidae Krzemiński and Krzemiński^3^, occurring near the early\middle Triassic boundary. The greatest radiation of these insects was during the Jurassic. The family Limoniidae Speiser^4^ are known since the Late Triassic, had become numerous among fossils from Europe^5–7^ and Asia^8,9^ since Early Jurassic (Toarcian). In the Middle Cretaceous, not only Triassic and Jurassic Limoniidae lineages are known, but also the representative of extant genera like *Helius* Lepeletiere & Sterville^10^ or *Dicranoptycha* Osten-Sacken^11^. Those known from fossil resins and sedimentary deposits of the Cretaceous and early Cenozoic^12,13^ are relatively numerous. Recently, this family comprised ca. 11.000 species^14^. Flies belonging to the extant subfamily Limoniinae were already present in the Cretaceous Period.

**Figure 1:**
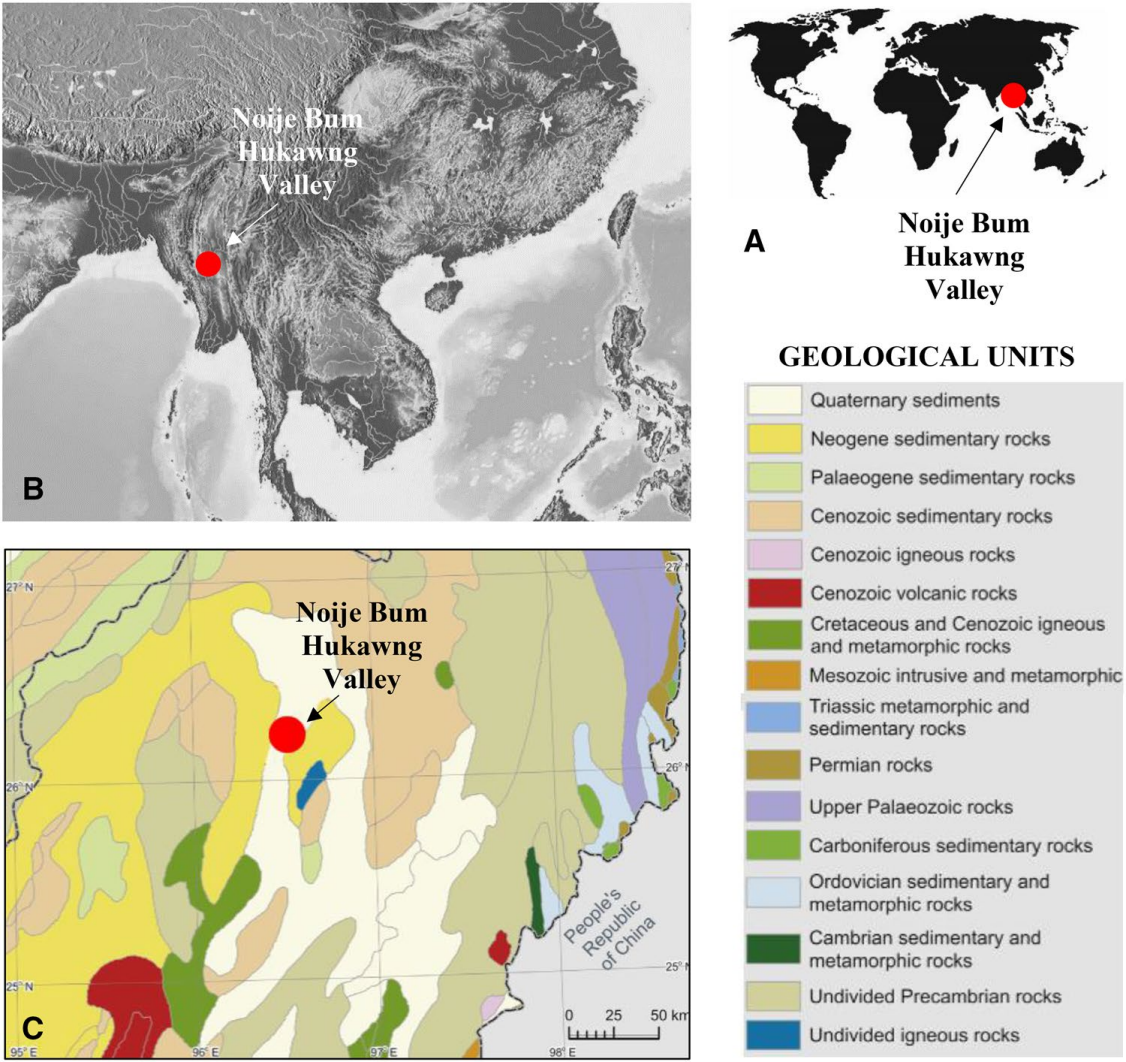
Maps of location of recent amber mining area in the Hukawng Valley, Myitkina Province, Burma. (**A**) Map of the world with location of Hukawng Valley; (**B**) Enlarged view of location of Hukawng Valley. (**C**). Geological setting of the Kachin amber deposits, after Kania^41^, modified. Maps were built using the map Maps-For-Free (https://maps-for-free.com) and modified with the software programs Corel Draw and Corel Photopaint X7.

The genus *Trichoneura* Loew^15^ is represented by 19 species (six extinct, 13 extant), divided into four subgenera: *Ceratolimnobia* Alexander^16^, *Cretalinea* Kania-Kłosok, Krzemiński, Kopeć & Arillo^17^, *Trichoneura* Loew^15^ and *Xipholimnobia* Alexander^18^. Two of them, *Cretalinea* and *Trichoneura*, are known from the fossil record. *Cretalinea* is known only from one Cretaceous species, *Trichoneura* (*Cretalinea*) *xavieri* Kania-Kłosok, Krzemiński, Kopeć, Arillo^17^. It was described from the Cretaceous (upper Albian) Peñacerrada I Basque—Cantabrian Basin, near the village of Moraza, Province of Burgos (Spain) and so far has been the oldest representative of the genus. The second species is known from the Cretaceous Period, *Trichoneura canadensis* Krzemiński et Teskey^19^, came from Upper Cretaceous amber from an open pit coal mine near Medicine Hat in southern Alberta, Canada and until now was treated as a representative of the subgenus *Trichoneura*. The subgenus *Trichoneura* is known mainly from Eocene amber (four species). From the recent fauna only one species, *Trichoneura* (*Trichoneura*) *umbrosa* Alexander^20^, is known and occurs in Australia and Oceania. The subgenus *Ceratolimnobia* is represented by two recent species: *Trichoneura* (*Ceratolimnobia*) *ishigakiensis* Kato^21^ and *Trichoneura* (*Ceratolimnobia*) *munroi* (Alexander^16^). There are ten recent species within the subgenus *Xipholimnobia* (Table 1). *Ceratolimnobia* and *Xipholimnobia* mainly occur in the Oriental region, but also in Cameroon, Nigeria and Madagascar^14^. The occurences of *Trichoneura* in Canadian amber, Baltic amber, Spanish and Kachin amber (described herein) (Fig. 1, Table 2) suggest that they were widely distributed in the past. Although, the subgenus *Trichoneura* is very rare in recent fauna, though from the fossil record six species are known. The study of new material preseved in Kachin amber provides additional information on the distribution and diversity of species of *Trichoneura*. The genus *Trichoneura* was probably not only widely distribiuted in the past but also numerous in species. The differentiated morphology and visible trends (reduction of huge lobe on gonocoxite and atrophy of R_3+4_). of the changing morphological features indicate two evolutionary branches—one extinct and one extant, but almost relict in the recent fauna. Based only on the morphology of wing venation it is possible to separate these two branches. The Cretaceous Period is very important for understanding the history of life on Earth and the evolution of modern ecosystems. Inclusions in Cretaceous resins, such as Barremian Lebanese amber (120–135 Ma)^22,23^, upper Albian Spanish amber (105 Ma)^24^, Albian French amber (Charente-Maritime, SW France)^25,26^ or younger Cenomanian Kachin amber (98.79 ± 0.62 Ma)^27^ document diversity and disparity of the World’s terrestrial fauna from over 98 milion years ago. There are other rich Cretaceous deposits, e.g. in China, like Late Cretaceous Xixia of Henan in Henan Province, Upper Cretaceous Jiayin amber in Heilongjiang Province^28^ or Hailar amber, the oldest known amber in China^29^, located within the Central Asian Orogenic Belt between the Siberian and North China–Mongolian cratons^30^. It was proposed that amber discovered from Lower Cretaceous deposits would bridge gaps among several well-known amber deposits, including Lebanese amber and Spain amber, amber from France or from Myanmar^29^.

**Table 1:** A list of recent species of the genus *Trichoneura* and their biogeographical distributions.

Species	Region	Country
*Ceratolimnobia*		
*Trichoneura* (*Ceratolimnobia*) *ishigakiensis* Kato^21^	Oriental	Japan
*Trichoneura* (*Ceratolimnobia*) *munroi* (Alexander^16^)	Afrotropic	Madagascar, Namibia, Nigeria, South Africa, Tanzania, Zimbabwe
Trichoneura*		
*Trichoneura* (*Trichoneura*) *umbrosa* Alexander^20^	Autralian and Oceanian	Indonesia
*Xipholimnobia*		
*Trichoneura* (*Xipholimnobi*) *bontocensis* Alexander^31^	Oriental	Philippines
*Trichoneura* (*Xipholimnobia*) *formosensis* (Alexander^32^)	Oriental	Taiwan
*Trichoneura* (*Xipholimnobia*) *jacksoni* Boardman^33^	Afrotropic	Cameroon
*Trichoneura* (*Xipholimnobia*) *japonica* Kato^21^	Oriental	Japan
*Trichoneura* (*Xipholimnobia*) *javanensis* Alexander^34^	Oriental	Indonesia
*Trichoneura* (*Xipholimnobia*) *madagascariensis* (Alexander^35^)	Afrotropic	Madagascar
*Trichoneura* (*Xipholimnobia*) *madrasensis* (Alexander^36^)	Oriental	India
*Trichoneura* (*Xipholimnobia*) *nepalensis* (Brunetti^37^)	Oriental	Nepal
*Trichoneura* (*Xipholimnobia*) *terebrina* (Alexander^18^)	Afrotropic	Cameroon, Nigeria
*Trichoneura* (*Xipholimnobia*) *umbripennis* Alexander^38^	Oriental	India

The subgenus marked with an asterisk (*) is also represented in the fossil record.

**Table 2:** List of fossils known belonging to the genus *Trichoneura*, with their ages, localities and information about the holotypes.

Species	Age	Typeof fossil resin	Locality	Sex	The number of holotype	Deposit of holotype
*Trichoneura* (*Trichoneura*) *gracilistylus* Alexander^39^	Eocene/Priabonian	Baltic amber	Baltic area	♂	No. 25	Coll. Klebs; GMUG
*Trichoneura* (*Trichoneura*) *ritzkowskii* Krzemiński^40^	Eocene/Priabonian	Baltic amber	Baltic area	♂	No. K5602 (Z16)	Coll. Klebs; GMUG
*Trichoneura* (*Trichoneura*) *wegiereki* Kania^41^	Eocene/Priabonian	Baltic amber	Baltic area	♂	No. MP 3447	ISEA PAS
*Trichoneura* (*Trichoneura*) *vulgaris* Loew^15^	Eocene/Priabonian	Baltic amber	Baltic area	♂	No. MB.J 350	Coll. Berendt; NHMB
*Trichoneura* (*Burmania*) *canadensis* Krzemiński et Teskey^19^ comb. nov	Upper Cretaceous/Campanian	Canadian amber	Open pit coal mine near Medicine Hat in southern Alberta	♂	No. 19078 in a piece of Canadian amber No. 1049	Canadian National Collection, (Ottawa)
*Trichoneura* (*Burmania*) *burmitensis* subgen. et sp. nov	Upper Cretaceous/Cenomanian	Kachin amber (Myanmar)	Kachin (Myanmar)	♂	No. MP/4365*	ISEA PAS
*Trichoneura* (*Burmania*) *chungkuni* subgen. et sp. nov	Upper Cretaceous/Cenomanian	Kachin amber (Myanmar)	Kachin (Myanmar)	♂	No. MP/4334	ISEA PAS
*Trichoneura* (*Burmania*) *sevciki* subgen. et sp. nov	Upper Cretaceous/Cenomanian	Kachin amber (Myanmar)	Kachin (Myanmar)	♂	No. BA02-851	Coll. B. WangNIGP
*Trichoneura* (*Burmania*) *wangi* subgen. et sp. nov	Upper Cretaceous/Cenomanian	Kachin amber (Myanmar)	Kachin (Myanmar)	♂	No. MP/4337	ISEA PAS
*Trichoneura* (*Cretalinea*) *xavieri* Kania-Kłosok, Krzemiński, Kopeć, Arillo^17^	Lower Cretaceous/upper Albian	Spanish amber	Peñacerrada I (Basque—Cantabrian Basin, near the village of Moraza, Province of Burgos)	♂	NMCNA 9735	Museo de Ciencias Naturales de Álava, (Vitoria, Spain)

## Results

Systematic palaeontology.

**Order: Diptera** Linnaeus^42^.

**Infraorder: Tipulomorpha** Rohdendorf^43^.

**Family: Limoniidae** Speiser^4^.

**Subfamily: Limoniinae** Speiser^4^.

**Genus: *Trichoneura*** Loew^15^.

**Subgenus: *Burmania*** subgen. nov.

Type-species*: Trichoneura* (*Burmania*) *burmitensis* subgen. et sp. nov.

LSID urn:lsid:zoobank.org:act:79247CE1-D8C3-4123-93EE-848A78002749.

*Diagnosis.* Vertex smooth, without corniculus; vein R_4_ separating from R_2+3+4_ far beyond separation of vein R_2_ (r-r), and with vein R_3_ forming sector R_3+4_.

*Etymology.* The specific epithet is derived from Burma (Myanmar).

*Description.* Body 3.08–3.84 mm long, brown, pterostigma sometimes present.

Head with antenna 16-segmented, 0.70–0.98 mm long, shorter than head and thorax combined; scape elongate, cylindrical; pedicel elongate, longer than wide, slightly wider than flagellomeres, flagellomeres oval elongate, at most twice as long as wide; becoming progressively slender toward antennal tip; last flagellomere usually shorter than penultimate one; the length of antennomeres according to: 1/0.10–0.15; 2/0.06–0.10; 3/0.05–0.08; 4/0.04–0.07; 5–16/0.04–0.05). Antenna with two–four moderately elongate setae on each flagellomeres; palpus four-segmented, slender, 0.22–0.31 mm long (1/0.07–0.08; 2/0.04–0.06; 3/0.04–0.06; 4/0.06) first, second and fourth palpomeres not very elongate, at least 3 × as long as wide, second palpomere sometimes widened in distal part, third palpomere sometimes widened in midlength.

Thorax: wing 2.94–5.00 mm long, 0.72–1.15 mm wide; R_3_ variable in length; R_4_ from one and a half to twice the length of d-cell; d-cell 0.27–0.46 mm long, approximately twice to twice and a half as long as wide; crossvein r-m usually elongate, equal or longer than basal section of R_5_; M_3_ shorter than M_1+2_, longer than M_4_; A_1_ and A_2_ elongate, usually almost straight, sometimes slighlty curved at the tip. Tergite IX with straight or only slightly indented front edge.

Abdomen with hypopygium 0.40–0.53 mm long; gonocoxite 0.25–0.38 mm long; outer gonostylus 0.07–0.19 mm long, inner gonostylus 0.17–0.22 mm long, aedeagus about 0.31 mm long.

*Comparison.* In *Cretalinea* gonocoxite is elongate, over 3 × as long as wide with huge, spoon-shaped lobe at apex measuring approximately 0.5 × the length of gonocoxite; gonostylus measuring less than 0.5 × the length of gonocoxite in *Burmania* subgen. nov. this lobe does not occur, gonocoxite is differentiated in length, gonostylus measuring more than 0.5 × the length of gonocoxite. Moreover, in *Burmania* subgen. nov. vein R_4_ separating from R_2+3+4_ far beyond separation of vein R_2_ (r–r), and with vein R_3_ forming sector R_3+4_; in *Trichoneura*, *Cretolimnobia* and *Xipholimnobia* vein R_4_ separates from R_2+3+4_ before or at the same point of separation of vein R_2_ (r–r), and R_3+4_ does not occur. In *Ceratolimnobia* occur corniculus on vertex and gonostylus is deeply bifid, in *Burmania* subgen. nov. vertex is smoth and gonostylus is undivided.

### New nomenclatoral decision

*Trichoneura* (*Trichoneura*) *canadiensis* Krzemiński and Teskey^19^ is transfered to the new subgenus *Burmania* subgen. nov. as *Trichoneura* (*Burmania*) *canadiensis* Krzemiński and Teskey^19^ comb. nov.

*Remark*: Such features as smooth vertex, without the cornicuus, presence of vein R_3+4_ and morphology of hypygium without huge, spoon-shaped lobe on its tip allow to classify this species to the new subgenus.

**Figure 2:**
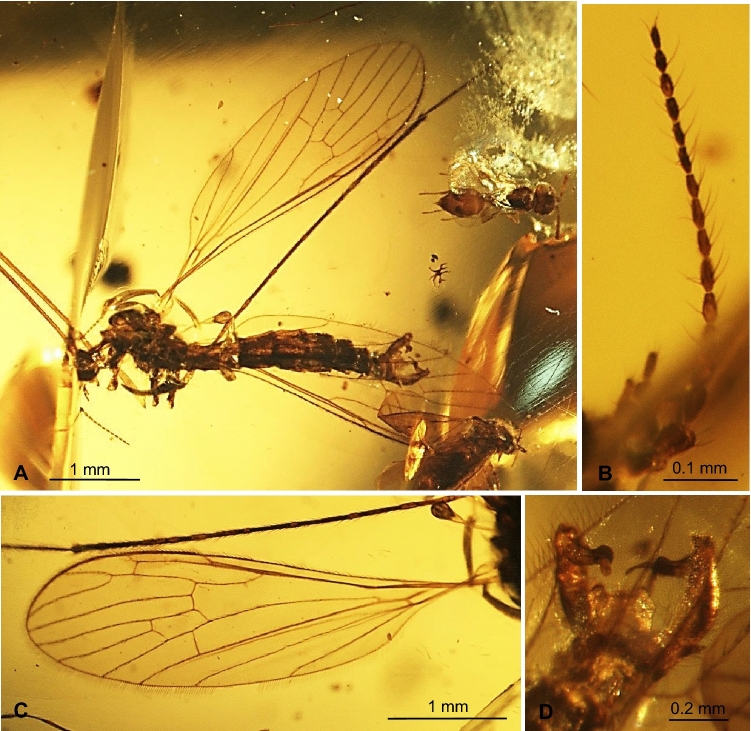
*Trichoneura* (*Burmania*) *burmitensis* subgen. et sp. nov. No. MP/4365, holotype (male) (ISEA PAS): (**A**) body, latero-dorsal view; (**B**) antenna and palpus; (**C**) wing; (**D**) hypopygium, dorsal view.

**Figure 3:**
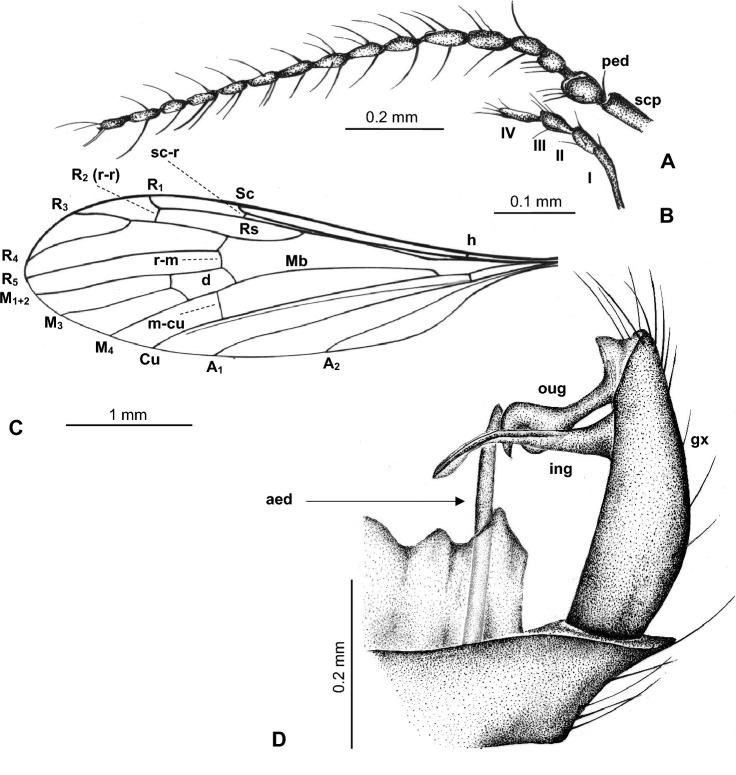
*Trichoneura* (*Burmania*) *burmitensis* subgen. et sp. nov. No. MP/4365, holotype (male) (ISEA PAS): (**A**) antenna; (**B**) palpus; (**C**) wing; (**D**) hypopygium, dorsal view. scp—scapus; ped—pedicel; I–IV—palpomeres 1–4; aed—aedeagus; gx—gonocoxite; ing—outer gonostylus; ing—inner gonostylus.

***Trichoneura* (*Burmania*) *burmitensis*** subgen. et sp. nov. (Figs. 2, 3).

LSID urn:lsid:zoobank.org:act:B7E1A8D2-FCF8-48E6-B045-D29487DA559C.

*Diagnosis.* Tip of Sc situated just before fork of Rs, sc-r at two of its length from the tip of Sc; vein R_1_ terminates at C opposite approximately 0.8 × common length of R_2+3+4_ and R_3+4_, tip of R_1_ curved; R_3+4_ slightly longer than R_2_ (r–r); R_5_ widely separated from Rs, basal section of R_5_ equal in length to r-m; m-cu just before midlength of d-cell; d-cell approximately twice as long as wide; tip of Cu beyond d-cell; tip of A_1_ beyond m-cu; tip of A_2_ situated opposite approximately half the length of Mb, medial-basal vein; gonocoxite not elongate, at most 2.5 × as long as wide with few, not very elongate setae at apex; outer gonostylus strongly curved, narrow in basal part, widened and sclerotized just before apex, apex of outer gonostylus narrow, pointed, with a brush of very short and coarse bristles at the end; inner gonostylus narrow, slightly sclerotized with narrow, pointed apex, inner gonostylus only approximately 0.3 × longer than outer; aedeagus thick, almost as long as gonocoxite, curved at apex.

*Etymology.* The specific epithet is derived from the Burmite.

*Type material. Holotype* No. MP/4365 (male) ISEA PAS; specimen in Kachin amber, Myanmar; *Paratypes* No. MP/4332 (male), No. MP/4335 (male), No. MP/4336 (male), No. MP/4340 (male) ISEA PAS, specimens in Kachin amber, Myanmar.

*Horizon and locality*. Lowermost Cenomanian, Hukawng Valley, northern Myanmar. The mining is done at a hill named Noije Bum, near Tanai Village (26° 21′ 33.41″ N, 96° 43′ 11.88″ E).

*Description.* Body (Figs. 2A, 3A–C) brown, 3.16–4.40 (holotype: 3.84) mm long, pterostigma present.

Head (Fig. 2A) with antenna (Figs. 2B, 3A) 0.97 mm (holotype) long (1/0.12; 2/0.10; 3/0.08; 4/0.07; 5/0.05–16/0.05), shorter than head and thorax combined; scape elongate, cylindrical, narrower than other segments of antenna; pedicel elongate, longer than wide, slightly wider than flagellomeres, flagellomeres oval elongate, approximately twice as long as wide; becoming progressively slender toward antennal tip; last flagellomere shorter than penultimate one. Antenna with three moderately elongate setae on each flagellomere, two on one side and one on the opposite side of each member; palpus (Figs. 2B, 3B) 0.22 mm (holotype) long (1/0.08; 2/0.04; 3/0.04; 4/0.06) first, second and fourth palpomeres not very elongate, approximately 3 × as long as wide, second palpomere widened in distal part, third palpomere widened in midlength.

Thorax (Fig. 2A): wing 2.94–5.00 mm long, 0.91–1.15 mm wide (holotype: 4.01 mm long, 1.00 wide) (Figs. 2A,C, 3C); tip of R_3_ beyond half the length of R_4_; R_4_ approximately twice the length of d-cell; d-cell 0.27–0.44 mm long (holotype), approximately twice as long as wide; crossvein r-m rather elongate, equal in length to basal section of R_5_; M_3_ 0.82 mm long (holotype), shorter than M_1+2_, longer than M_4_; A_1_ and A_2_ elongate, almost straight.

Abdomen (Fig. 2A): hypopygium wide (Figs. 2A,D, 3D), 0.41–0.46 mm long (holotype); gonocoxite 0.25–0.33 mm long (holotype); outer gonostylus 0.16 (holotype) − 0.19 mm long, inner gonostylus 0.19 mm long (holotype), aedeagus 0.31 mm long.

*Comparison.* In *Trichoneura* (*Burmania*) *burmitensis* subgen. et sp. nov. the tip of Sc is situated just before the fork of Rs, and sc-r at two of its length from the tip of Sc, in *T.* (*B.*) *wangi* the tip of Sc is situated opposite approximately 0.8 × before the fork of Rs, and vein sc-r at one of its length from the tip of Sc. Moreover, vein R_1_ in *T.* (*B.*) *burmitensis* terminates at C opposite approximately 0.8 × common length of R_2+3+4_ and R_3+4_, tip of R_1_ is curved, R_3+4_ is slightly longer than R_2_ (r–r), while in *T.* (*B.*) *wangi* vein R_1_ terminates at C opposite approximately 0.9 × common length of R_2+3+4_ and R_3+4_, tip of R_1_ is straight, R_3+4_ is slightly shorter than R_2_ (r-r). In *T.* (*B.*) *burmitensis* R_5_ is widely separated from Rs, basal section of R_5_ is equal in length to r-m, in *T.* (*B.*) *wangi* R_5_ is narrowly separated from Rs, basal section of R_5_ is shorter in length to r-m. There are also some differences in the position of tips Cu, A_1_ and A_2_. In *T.* (*B.*) *burmitensis* tip of Cu is situated beyond d-cell level, tip of A_1_ beyond m-cu level and tip of A_2_ opposite approximately half the length of Mb, in *T.* (*B.*) *wangi* tip of Cu is situated at d-cell level, tip of A_1_ before m-cu level and tip of A_2_ opposite approximately 0.3 × the length of Mb. But, the main differences are visible in the shape of outer gonostylus: in *T.* (*B.*) *burmitensis* subgen. et sp. nov. this structure is narrow at the basal part, widened just before apex, in *T.* (*B.*) *wangi* outer gonostylus is wide along its entire length, tiped at apex. In contrast to *T.* (*B.*) *chungkuni*, in *T.* (*B.*) *burmitensis* gonocoxite is not elongate, at most 2.5 × as long as wide with only few, not very elongate setae at apex, aedeagus is thick, almost as long as gonocoxite, curved at apex, in *T.* (*B.*) *chungkuni* gonocoxite is elongate, at least 3.5 × as long as wide, aedeagus is distinctly shorter than gonocoxite, approximately 0.6 × of its length. In *T.* (*B.*) *sevciki*, aedeagus is longer than gonocoxite, outer gonostylus is rather narrow and inner gonostylus is elongate and lobe shaped, widened at apex, in *T.* (*B.*) *burmitensis* outer gonostylus is strongly curved, widened and sclerotized just before apex, apex of outer gonostylus is narrow, pointed, inner gonostylus is narrow, slightly sclerotized with narrow, pointed apex. From Canadian amber *T.* (*B.*) *canadensis* comb. nov. inner gonostylus is long, twice longer than outer gonostylus, in *T.* (*B.*) *burmitensis* inner gonostylus is shorter.

***Trichoneura* (*Burmania*) *chungkuni*** subgen. et sp. nov.

**Figure 4:**
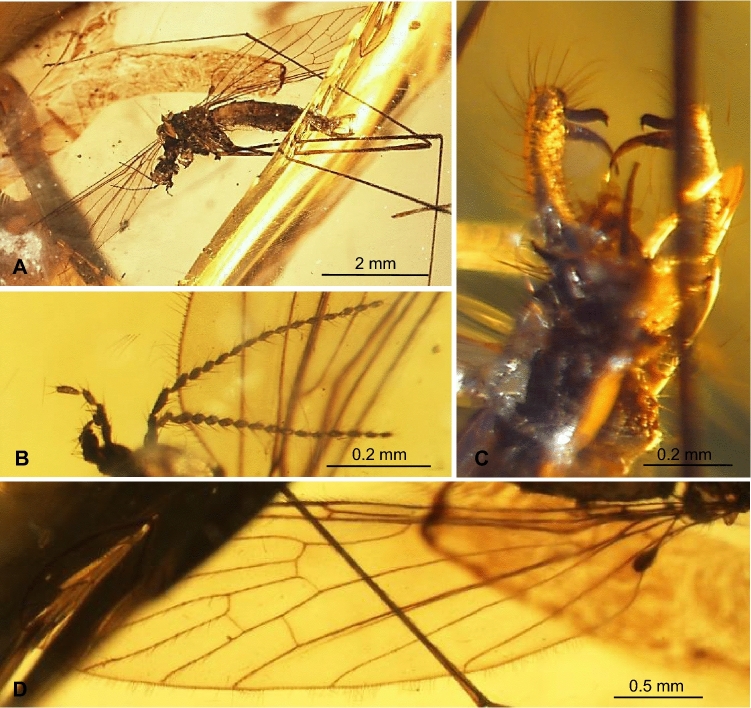
*Trichoneura* (*Burmania*) *chungkuni* subgen. et sp. nov. No. MP/4334, holotype (male) (ISEA PAS): (**A**) body, lateral view; (**B**) palpus and antenna; (**C**) hypopygium, ventral view; (**D**). wing.

**Figure 5:**
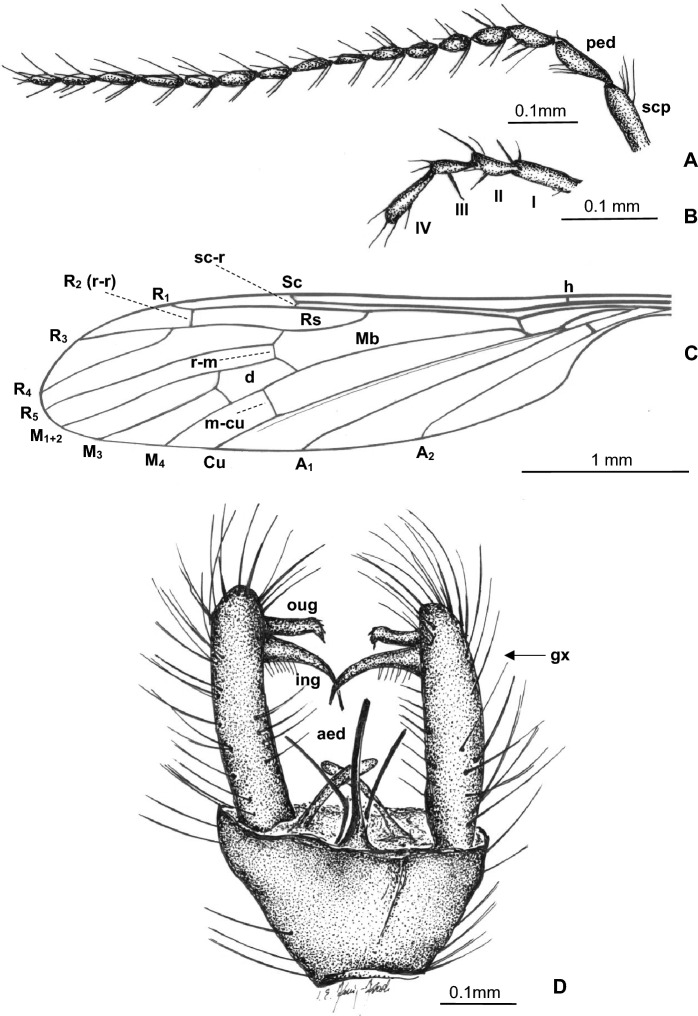
*Trichoneura* (*Burmania*) *chungkuni* subgen. et sp. nov. No. MP/4334, holotype (male) (ISEA PAS): (**A**) antenna; (**B**) palpus; (**C**) wing; (**D**) hypopygium, ventral view. Abbreviations as in Fig. 3.

LSID urn:lsid:zoobank.org:act:4BF9D26F-2A2E-4AB8-AEB4-144B8362FEB3. (Figures. 4, 5).

*Diagnosis.* Tip of Sc situated just before fork of Rs, sc-r at two of its length from the tip of Sc; vein R_1_ terminates at C opposite at fork of R_3+4_, tip of R_1_ almost straight; R_3+4_ shorter than R_2_ (r–r); R_5_ widely separated from Rs, basal section of R_5_ equal in length to r-m; m-cu at midlength of d-cell; d-cell approximately twice as long as wide; tip of Cu beyond d-cell level; tip of A_1_ before m-cu level; tip of A_2_ situated just before half the length of Mb, medial-basal vein; gonocoxite very elongate, at least 3.5 × as long as wide with numerous, dense, elongate and thick setae especially concentrated at apex; outer gonostylus tiny, pointed and curved at apex, twice longer than inner, inner gonostylus narrow, thick with brush of setae at apex, aedeagus tiny, not very elogate, 0.6 × the length of gonocoxite, not divided.

*Etymology.* The specific epithet is dedicated to Prof. Chungkun Shih (Key Lab of Insect Evolution and Environmental Changes, College of Life Sciences, Capital Normal University, Beijing 100,048, China), the eminent specialist on fossil and recent insects.

*Type material. Holotype* MP/4334 (male) ISEA PAS; specimen in Kachin amber, Myanmar. *Horizon and locality.* Lowermost Cenomanian, Hukawng Valley, northern Myanmar. The mining is done at a hill named Noije Bum, near Tanai Village (26° 21′ 33.41″ N, 96° 43′ 11.88″ E).

*Description.* Body (Fig. 4A) brown, 3.84 mm long.

Head (Fig. 4A) with antenna (Figs. 4A,B, 5A) 0.70 mm long (1/0.10; 2/0.08; 3/0.07; 4–16/0.05), shorter than head and thorax combined; scape elongate, cylindrical, as narrow as other segments of antenna; pedicel elongate, longer than wide, widened in midlength, flagellomeres oval, not very elongate, approximately twice as long as wide; becoming progressively slender toward antennal tip; last flagellomere only slightly shorter than penultimate one. Antenna with four moderately elongate setae on each flagellomeres, two on one side and two on the opposite side of each flagellomeres; palpus (Figs. 4A, B 5B) 0.31 mm long (1/0.07; 2–4/0.06) four-segmented, palpomeres not very elongate, but the last one as long as two penultimate; few setae on each palpomeres.

Thorax (Fig. 4A): wing 3.80 mm long, 0.94 mm wide (Figs. 4A,D, 5C); vein R_1_ elongate, ending opposite fork of R_3+4_ on R_3_ and R_4_; tip of R_3_ beyond half the length of R_4_; R_4_ approximately twice the length of d-cell; d-cell 0.46 mm long, approximately twice as long as wide; crossvein r-m rather elongate, equal in length to basal section of R_5_; M_3_ 0.81 mm long, shorter than M_1+2_, longer than M_4_; A_1_ and A_2_ elongate, A_1_ almost straight, A_2_ curved at the tip.

Abdomen (Fig. 4A): hypopygium narrow (Figs. 4A,C, 5D), 0.49 mm long; gonocoxite 0.35 mm long, 0.07 mm wide; outer gonostylus 0.11 mm long, inner gonostylus 0.22 mm long.

*Comparison.* The main difference between the *Trichoneura* (*Burmania*) *chungkuni* subgen. et sp. nov. and other species of *Burmania* subgen. nov. known from Cretaceous is the morphology of hypopygium. Hypopygium of *T.* (*B.*) *chungkuni* is narrow, with elongate, at least 3.5 × as long as wide. Gonocoxite with dense, elongated setae, especially at the tip of gonocoxite with aedeagus not very elongate, reaching at most 0.6 × the length of gonocoxite and lobe shaped inner gonostylus. In other species of *Burmania* the hypopygium is wide with not very elongate gonocoxite, similarly to these species known from Baltic amber, aedeagus is almost as long as gonocoxite or longer and inner gonostylus is narrow and tipped. Some differences are well visible in wing venation. R_1_ in *T.* (*B.*) *chungkuni* it is ending opposite fork of R_3+4_ on R_3_ and R_4_, while in other species of *Burmania* is also elongate, but always ended before this bifurcation.

***Trichoneura* (*Burmania*) *sevciki*** subgen. et sp. nov.

**Figure 6:**
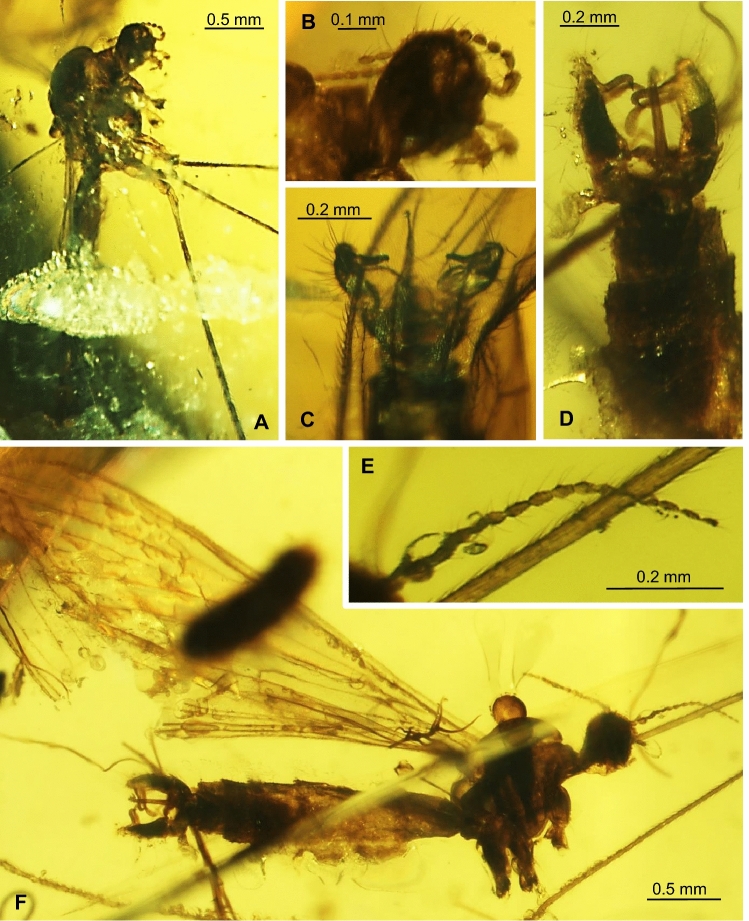
*Trichoneura* (*Burmania*) *sevciki* subgen. et sp. nov. No. NIGP177895, holotype (male) (**A**–**C**): (**A**) body, lateral view; (**B**) head with antenna and palpus visible; (**C**) hypopygium, ventral view; No. 52/2019, paratype (male) (ISEA PAS), paratype (male) (**D**–**F**): hypopygium, ventral view; (**E**) antenna; (**F**) body, lateral view.

**Figure 7:**
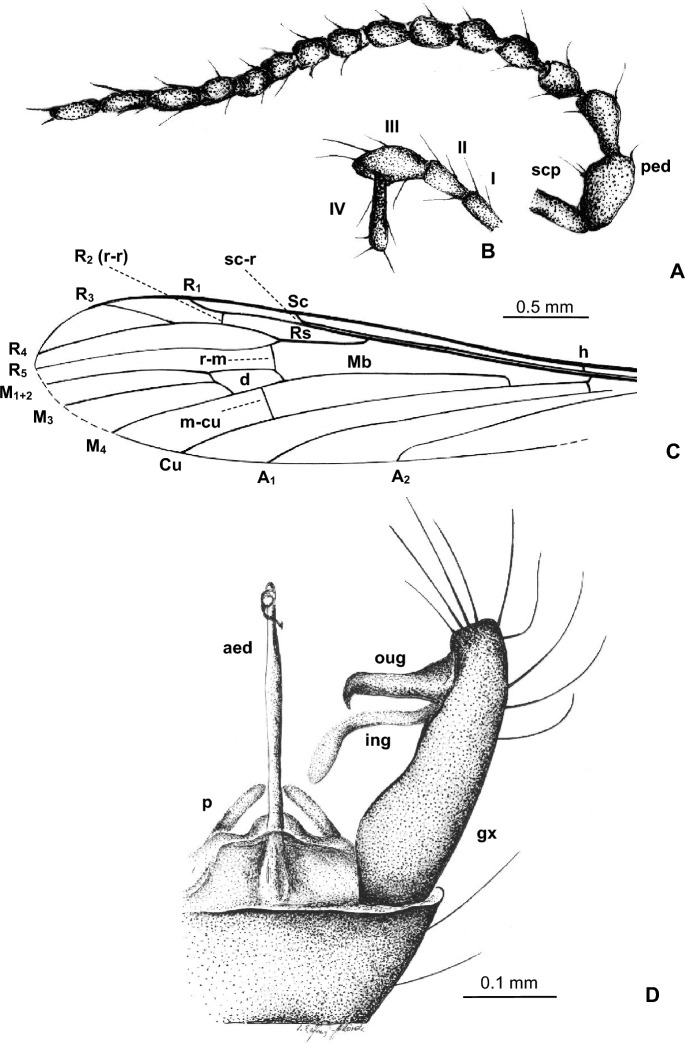
*Trichoneura* (*Burmania*) *sevciki* subgen. et sp. nov. No. NIGP177895, holotype (male) (NIGP). (**A**) antenna, reconstruction; (**B**) palpus; (**C**) wing; (paratype No. 52/2019, paratype (male) (ISEA PAS); (**D**) hypopygium, ventral view. Abbreviations as in Fig. 3.

LSID urn:lsid:zoobank.org:act:0BAC90FF-9210-4AE9-90A2-9E72D3FD6AF9. (Figures. 6, 7).

*Diagnosis.* Vein R_1_ terminates at C opposite approximately 0.8 × common length of R_2+3+4_ and R_3+4_, tip of R_1_ curved; R_3+4_ longer than R_2_ (r–r); R_5_ widely separated from Rs, basal section of R_5_ shorter than r-m; m-cu before midlength of d-cell; d-cell approximately twice as long as wide; tip of Cu far beyond d-cell level; tip of A_1_ before m-cu level; tip of A_2_ situated opposite approximately 0.5 × the length of Mb, medial-basal vein; gonocoxite elongate, approximately 3 × as long as wide, with few, not very elongate setae at apex; aedeagus with extension and curved, elongate appendix at apex, longer than gonocoxite; inner gonostylus elongate, lobe shaped, only slightly sclerotized, widened in distal part, rounded, outer gonostylus tiped at apex, rather straight, arrange 0.5 × length of inner gonostylus.

*Etymology.* The specific epithet is dedicated to Dr. Jan Ševčík Department of Biology and Ecology, Faculty of Science, University of Ostrava, the eminent specialist on fossil and recent insects.

*Type material. Holotype* No. NIGP177895 (male) NIGP, coll. B. Wang, specimen in Kachin amber, Myanmar; *Paratype* No. 52/2019 (male) ISEA PAS, coll. J. Ševčík.

*Horizon and locality.* Lowermost Cenomanian, Hukawng Valley, northern Myanmar. The mining is done at a hill named Noije Bum, near Tanai Village (26° 21′ 33.41″ N, 96° 43′ 11.88″ E).

*Description.* Body (Fig. 6A,F) brown, 3.08–3.72 (holotype) mm long.

Head (Fig. 6A,B) with antenna (Figs. 6A,B,E, 7A) 0.64 mm long, shorter than head and thorax combined; scape elongate, narrow, cylindrical, longer than pedicel, wider than other segments of antenna; pedicel elongate, longer than wide, widened, approximately as long as first flagellomere; flagellomeres wide, approximately as wide as long, but becoming progressively slender toward antennal tip; first flagellomere elongate, longer than the rest, approximately 4 × as long as wide; last flagellomere longer than penultimate one. Antenna with two moderately elongate setae on each flagellomeres, one on one side and one on the opposite side of each member; palpus (Figs. 6A,B, 7B) four-segmented, first, second and fourth palpomeres narrow, sleder, third palpomere widened distally, last palpomere longer than rest, all palpomeres with few, rather not very elongate setae, shorter than segments bearing them.

Thorax (Fig. 6A,F): wing (Figs. 6F, 7C) 3.57 mm long, 0.89 mm wide; vein R_1_ rather elongate, ending opposite 0.5 × length of vein R_3+4_; d-cell approximately twice as long as wide; A_1_ and A_2_ elongate, slightly curved at the tip.

Abdomen (Figs. 6A,F, 7D,C): hypopygium wide (Figs. 6C,D, 7D), 0.40–0.52 mm long (holotype).

*Comparison.* The most characteristic feature which separates *Trichonerua* (*Burmania*) *sevciki* subgen. et sp. nov. from all known Cretaceous species of *Trichoneura* is morphology of gonostyles and aedeagus, aedeagus is very elongate, longer than gonocoxite with extension, curved, elongate appendix at apex, lobe shaped and only slightly sclerotized inner gonostylus.

***Trichoneura* (*Burmania*) *wangi*** subgen. et sp. nov.

**Figure 8:**
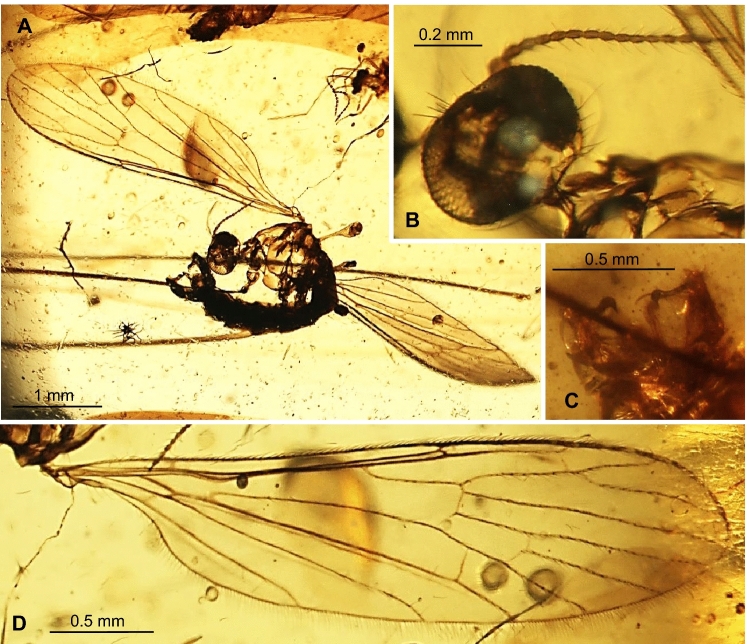
*Trichoneura* (*Burmania*) *wangi* subgen. et sp. nov. No. MP/4337, holotype (male) (ISEA PAS): (**A**) body, lateral view; (**B**) head with antenna visible, dorsal view; (**C**) hypopygium, ventral view; (**D**) wing.

**Figure 9:**
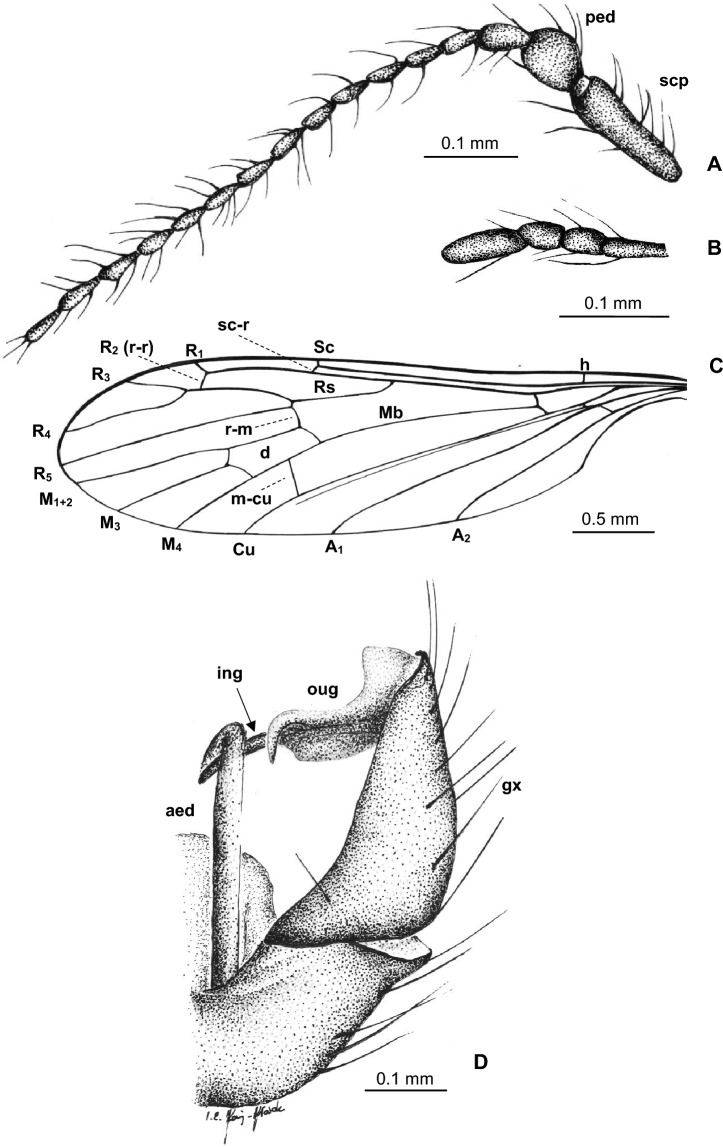
*Trichoneura* (*Burmania*) *wangi* subgen. et sp. nov., No. MP/4337, holotype (male) (ISEA PAS): (**A**) antenna; (**B**) palpus; (**C**) wing; (**D**) hypopygium, ventral view. Abbreviations as in Fig. 3.

LSID urn:lsid:zoobank.org:act:5985861F-7EE9-4722-9FAF-E35A1BDC4A00. (Figs. 8, 9).

*Diagnosis.* Tip of Sc situated just before fork of Rs, sc-r at one of its length from the tip of Sc; vein R_1_ terminates at C opposite approximately 0.9 × common length of R_2+3+4_ and R_3+4_, tip of R_1_ curved; R_3+4_ shorter than R_2_ (r–r); R_5_ narrowly separated from Rs, basal section of R_5_ shorter than r-m; m-cu in midlength of d-cell; d-cell approximately twice as long as wide; tip of Cu at d-cell level; tip of A_1_ before m-cu level; tip of A_2_ situated opposite approximately 0.3 × the length of Mb, medial-basal vein; gonocoxite with few, not very elongate setae at apex; outer and inner gonostyles almost equal in length, outer gonostylus broad and strongly curved at apex, pointed, slightly sclerotized, inner gonostylus narrow, pointed at apex, slightly folded, longer than outer gonostylus; aedeagus thick, elogate, curved at apex only slightly shorter than gonocoxite.

*Etymology.* The specific epithet is dedicated to Prof. Bo Wang (State Key Laboratory of Palaeobiology and Stratigraphy, Nanjing Institute of Geology and Palaeontology, Chinese Academy of Sciences, China), the eminent specialist on fossil and recent insects.

*Type material. Holotype* MP/4337 (male) ISEA PAS; specimen in Kachin amber, Myanmar.

*Horizon and locality.* Lowermost Cenomanian, Hukawng Valley, northern Myanmar. The mining is done at a hill named Noije Bum, near Tanai Village (26° 21′ 33.41″ N, 96° 43′ 11.88″ E).

*Description.* Body (Fig. 8A) brown, 3.34 mm long.

Head (Fig. 8A) with antenna (Figs. 8A, B, 9A) 0.78 mm long (1/0.15; 2/0.06; 3/0.05; 4–16/0.04), shorter than head and thorax combined; scape elongate, cylindrical, longer than other segments of antenna; pedicel buble-like, flagellomeres cylindrical, elongate, approximately 2 × as long as wide; becoming progressively slender toward antennal tip; last flagellomere tinner than penultimate one, but of similar length. Antenna with two moderately elongate setae on flagellomeres 1–6 and four on flagellomeres 7–13; palpus (Fig. 9B) four-segmented, palpomeres first, second and fourth not very elongate, approximately 3 × as long as wide, rather narrow only third palpomere widened in midlength.

Thorax (Fig. 8A): wing 3.72 mm long, 0.98 mm wide (Figs. 8A,D, 9C); vein R_1_ elongate, ending opposite approximately 0.9 × length of vein R_3+4_; tip of R_3_ at the midlength of R_4_; R_4_ 1.5 × the length of d-cell; d-cell 0.27. mm long, 2.5 × as long as wide; crossvein r-m short, shorter than basal section of R_5_; M_3_ 0.70 mm long, shorter than M_1+2_, longer than M_4_; A_1_ and A_2_ elongate, slightly waved.

Abdomen (Figs. 8A): hypopygium wide, 0.50 mm long; gonocoxite rather short; outer and inner gonostylus almost the same length (Figs. 8A,C, 9D).

*Comparison.* See comparison of *Trichoneura* (*Burmania*) *burmitensis* subgen. et sp. nov. above. Moreover, in contrast to *T.* (*B.*) *chungkuni*, in *T.* (*B.*) *wangi* gonocoxite is not elongate, at most 2.5 × as long as wide with only few, not very elongate setae at apex, in *T.* (*B.*) *chungkuni* gonocoxite is elongate, at least 3.5 × as long as wide. In contrast to *T.* (*B.*) *sevciki*, in *T.* (*B.*) *wangi* aedeagus is thick, not very elogate, no longer than gonocoxite, while in *T.* (*B.*) *sevciki* aedeagus is longer than gonocoxite. In contrast to *T.* (*B.*) *canadensis* comb. nov. where inner gonostylus is long, twice longer than outer gonostylus, in *T.* (*B.*) *wangi* inner gonostylus is shorter.

### Key to species of *Burmania* subgen. nov.

1. Aedeagus shorter than gonocoxite, inner gonostylus narrow … 2.
  - Aedeagus longer than gonocoxite, inner gonostylus lobe shaped … ***Trichoneura* (*Burmania*) *sevciki*** subgen. et sp. nov. (Figs. 6C,D 7D).
2. Gonocoxite at most 2 × as long as wide, not very elongate setae on apex of gonocoxite shorter than half of its length; R_1_ always ended before fork of R_3+4_ on R_3_ and R_4_ … 3.
  - Gonocoxite at least 3.5 × as long as wide, very elongate setae on apex of gonocoxite longer than half of its length; R_1_ ending opposite fork of R_3+4_ on R_3_ and R_4_ … ***Trichoneura* (*Burmania*) *chungkuni*** subgen. et sp. nov. (Figs. 4C, 5D).
3. Vein sc-r at two of its length from the tip of Sc; vein R_1_ terminates at C opposite approximately 0.8 × common length of R_2+3+4_ and R_3+4_; R_3+4_ slightly longer than R_2_ (r-r); R_5_ widely separated from Rs; m-cu just before midlength of d-cell; tip of Cu beyond d-cell level; tip of A_1_ beyond m-cu level; tip of A_2_ situated opposite approximately half the length of Mb; inner gonostylus narrow, lobe shaped, rounded at apex, slightly folded … ***Trichoneura* (*Burmania*) *burmitensis*** subgen. et sp. nov. (Figs. 2A,C, 3C).
  Vein sc-r at one of its length from the tip of Sc; vein R_1_ terminates at C opposite approximately 0.9 × common length of R_2+3+4_ and R_3+4_; R_3+4_ shorter than R_2_ (r-r); R_5_ narrowly separated from Rs; m-cu in midlength of d-cell; tip of Cu at d-cell level; tip of A_1_ before m-cu level; tip of A_2_ situated opposite approximately 0.3 × the length of Mb; gonocoxite with few, not very elongate setae at apex; outer gonostylus broad in distal part and strongly curved at apex, pointed, strongly sclerotized, inner gonostylus narrow, lobe shaped, pointed at apex; … ***Trichoneura* (*Burmania*) *wangi*** subgen. et sp. nov. (Figs. 8C,D, 9C).

## Discussion

**Figure 10:**
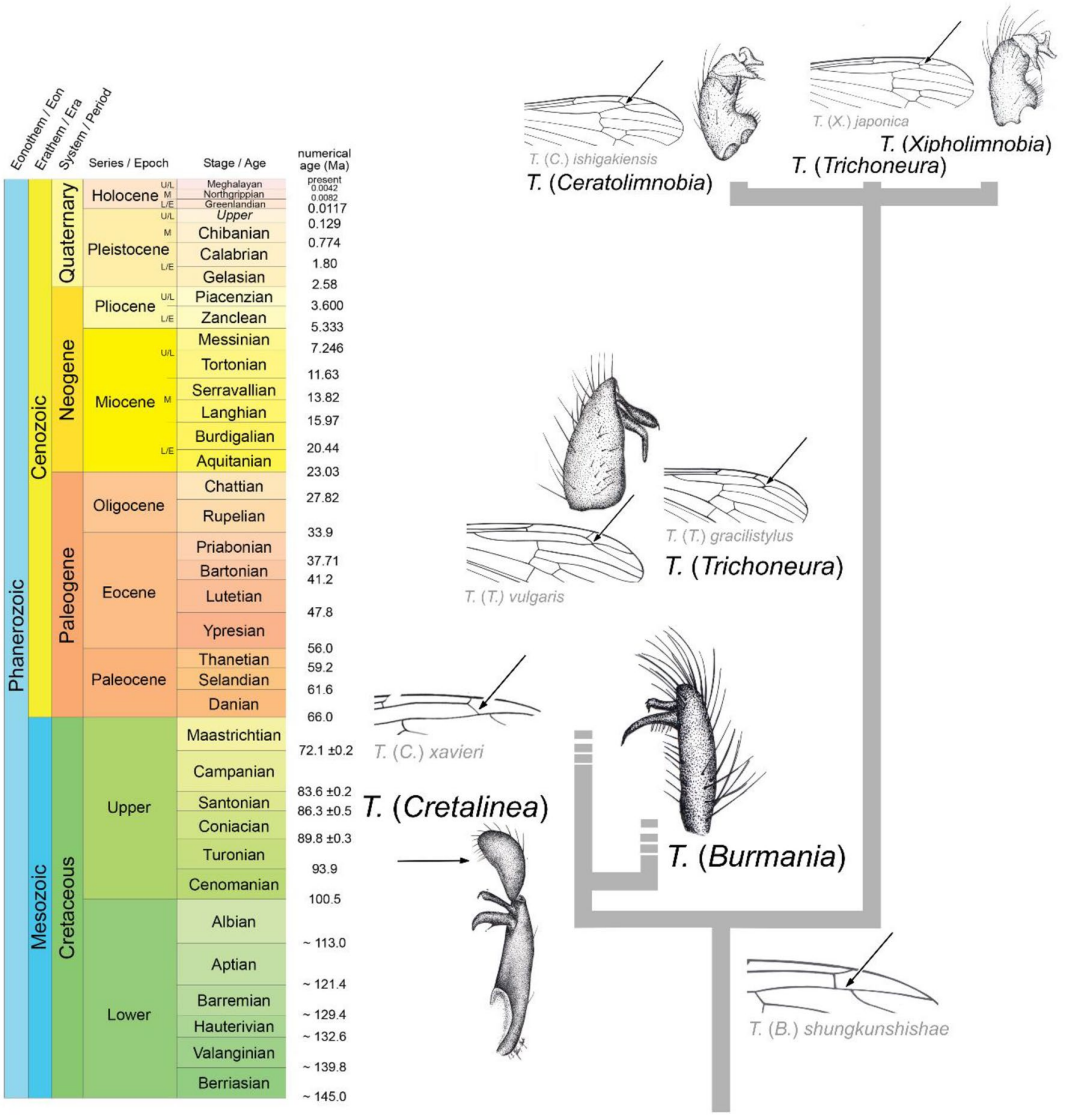
Diagram of potential lines of evolution within the genus *Trichoneura* in chronostratigraphical view with examples of wing venation and morphology of gonocoxites and gonostyles of chosen species of the genus.

The oldest representatives of *Trichoneura*—*T.* (*C.*) *xavieri* is from Lower Cretaceous Spanish amber. This species exhibits a unique morphology of its hypopygium characterized by a huge lobe on the gonocoxite^17^. The newly described herein new subgenus *Burmania* subgen. nov. (Table 2) characterize by the absence of lobe on gonocoxite, while wing venation of these insects indicates a close relationship with *Cretalinea*, both characterize by the well presented R_3+4_. Interestingly, in Eocene representatives of *Trichoneura* and those that occur in the recent fauna, vein R_2_ (r–r) is shifted toward the apex of wing, and is connected with fork of R_3+4_ on R_3_ and R_4_ or is even positioned beyond this fork. The hypopygium in the Eocene species of subgenus *Trichoneura* is rather wide (this feature is well visible). A huge lobe which occurred on the gonocoxite of upper Albian (Lower Cretaceous) *T.* (*C.*) *xavieri* was probably subsequently reduced and the apex of the gonocoxite was shortened, as seen in Eocene and recent species of this genus. In *Burmania* subgen. nov. the huge lobe on the gonocoxite is not presented. The Cretaceous line with a huge lobe on the gonocoxite or elongate gonocoxite is completely extinct (Fig. 10).

Analysis of morphological features of craneflies of the genus *Trichoneura* shows that two evolutionary branches were probably separated at the early stage of evolution of these insects. In the recent fauna this group is almost relict, represented by only 13 species within three subgenera. In the evolution of this group the evolutionary tendencies are visible, especially in the morphology of the wing venation (Fig. 10), whereby the radial vein R_1_ was gradually shortened. In Cretaceous representatives we can observe an elongate R_1_ which terminates far beyond half the length of Rs. Also, in Cretaceous representatives, such as *T.* (*C.*) *xavieri* from Spanish amber and in those described herein from Burmese amber under the new subgenus *Burmania* occurs a short vein R_3+4_, whereas in Eocene species vein R_3+4_ doesn’t occur.

The *Trichoneura* genus, dynamically developing in the Mesozoic, evolved mainly in what was then Laurasia.

**Figure 11:**
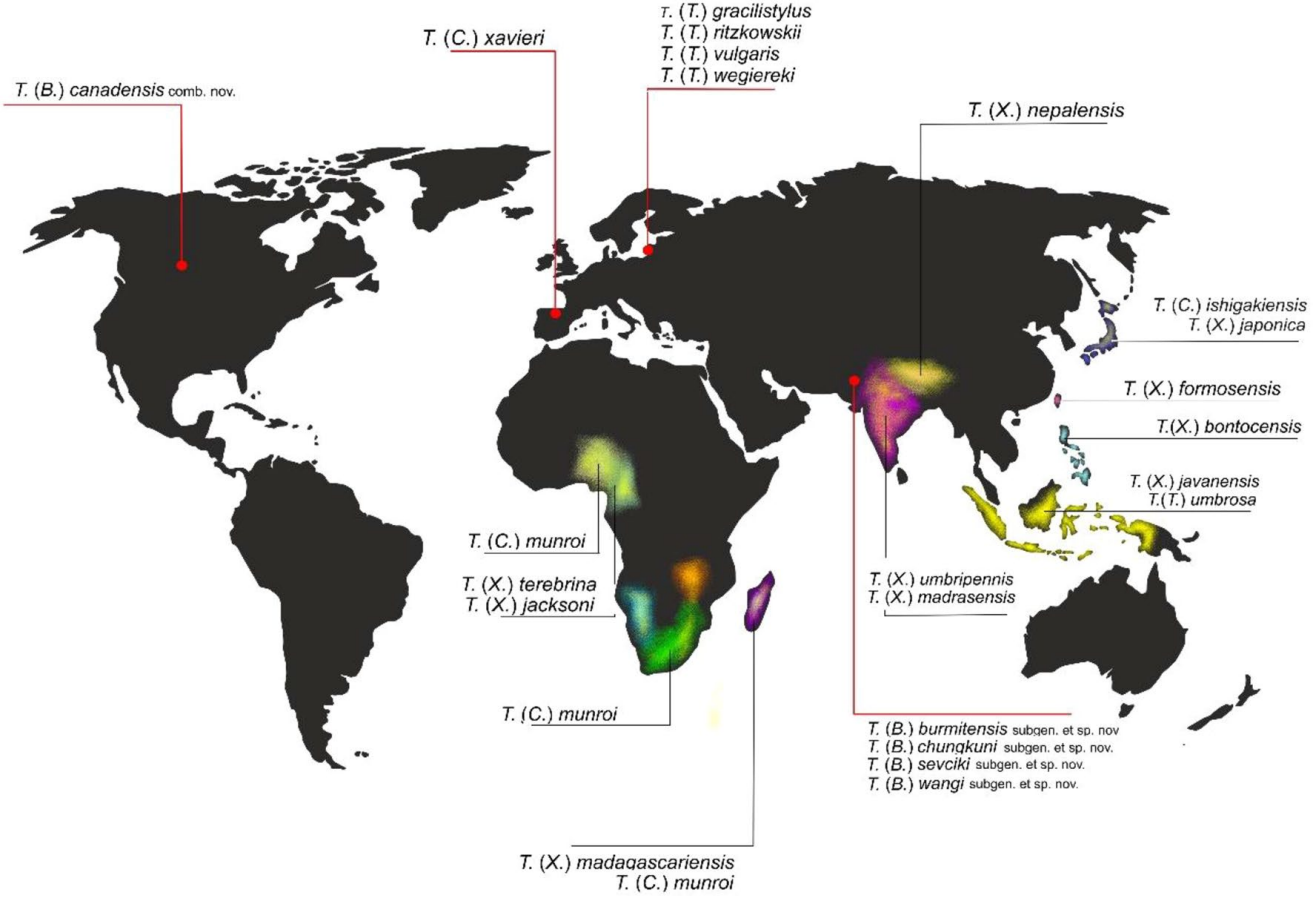
Geographical distribution of fossil and recent species of the genus *Trichoneura*. Points indicate fossil localities of *Trichoneura*, colour shadow—widespread of recent species of the genus. Map was built using the map Maps-For-Free (https://maps-for-free.com) and modified with the software programs Corel Draw and Corel Photopaint X7.

Evidence of its widespread occurrence in this subcontinent is found in the fossil resins of Europe^15,17,19,41^, Asia (the species described herein) and North America^40^ (Table 2). Unfortunately, we have no fossil evidence of the presence of representatives of *Trichoneura* from Gondwana in the Mesozoic. In the modern fauna only in Africa we find four representatives of the *Trichoneura* genus and several species in the Australian/Oceanian Region (Fig. 11). In modern fauna, representatives of the subgenus *Xipholimnobia* are more numerous in species than *Ceratolimnobia*, species belonging to both subgenera occur at similar latitiudes, e.g. *T.* (*C.*) *ishigakiensis* is found in Japan, as is *T.* (*X.*) *japonica*^21^, and *T.* (*C.*) *munroi* occur in Madagascar similarly to *T.* (*X.*) *madagascariensis*^14^. The presence of these modern, relict species may indicate the occurrence of representatives of the *Trichoneurana* genus also in Gondwana in past geological epochs.

Limoniidae are highly variable regarding their ecology and biology, their larvae are found in a wide spectrum of habitats, ranging from running waters, through still and stagnant ones, bottom sediments, to terrestrial habitats such as soils, litter, and detritus^44–47^. Unlike larvae, imagines of Limoniidae are more habitats restricted and usually present in shady and moist places, often near the shores and banks of waters, feeding on nectar and plant juices exuded on their surface^48–51^.

Palaeoentomological investigations on fossil Limoniinae demonstrated the existence of obstacles and needs to reinterpretation of biogeographic opinions concerning these flies. A better knowledge of fossil Limoniinae had enabled to provide palaeohabitats reconstructions and ecological interpretations of the past environments, in which these insects existed.

## Material and methods

The study was based on 10 inclusions of the genus *Trichoneura* (Limoniidae: Limoniinae) preserved in Cretaceous Kachin amber, aged on 98.79 ± 0.62 Ma, (Upper Cretaceous, Cenomanian)^2,52,53^. The specimens were found as inclusions in the deposits located at the Hukawng Valley in the northern Myanmar, Myitkyina and Upper Chindwin districts (Myanmar)^27,54^ (Fig. 1) and are housed in Institute of Systematics and Evolution of Animals, Polish Academy of Sciences, Kraków (ISEA PAS) (eight specimens) and in State Key Laboratory of Palaeobiology and Stratigraphy, Nanjing Institute of Geology and Palaeontology, Chinese Academy of Sciences, China, coll. B. Wang (one specimen) (Table 2). The specimens were examined using a Nikon SMZ 1500 stereomicroscope equipped with a Nikon DS-Fi1 camera, and the measurements were taken with NIS-Elements D 3.0 software in University of Rzeszów. Measurements of individual parts of the body were given only when the measured morphological structures were not distorted. The length of the vein M_3_ was given from the point of its connection with the crossvein m-m to the margin of wing, the length of the discal cell was given from its posterior edge to the point of connection of vein m-m with vein M_3_. The length of hypopygium was measured from the posterior margin of tergite IX to the apex of gonocoxite. Drawings were made based on specimens and the photographs. Drawings and photographs were made by Iwona Kania-Kłosok. The wing venation nomenclature and the designation of the hypopygium is followed by Kania^41^. Maps were built using the map Maps-For-Free (https://maps-for-free.com) and modified with the software programs Corel Draw and Corel Photopaint X7.

*Statement* The specimen (NIGP177895) involved in this study were collected in 2015. These specimens are now deposited in the Nanjing Institute of Geology and Palaeontology, Chinese Academy of Sciences. The collection and storage process of these specimens were in full compliance with the regulations of fossils specimen procurement of the institute. Access is free to all scientist permanently. The specimens (MP/4365, MP/4332, MP/4334, MP/4335, MP/4336, MP/4337, MP/4340) reported in this study were donated to the Natural History Museum of Institute of Systematics and Evolution of Animals Polish Academy of Sciences by the collector Mr. Jacek Serafin in 2001, the specimen No. 52/2019 by dr. Jan Ševčík. They will be permanently deposited in Natural History Museum of Institute of Systematics and Evolution of Animals Polish Academy of Sciences, Kraków, Poland (ISEA PAS).

## Data Availability

All data generated or analyzed during this study are included in this published article.
